# Association between C-reactive protein and chronic pain in US adults: A nationwide cross-sectional study

**DOI:** 10.1371/journal.pone.0315602

**Published:** 2025-02-07

**Authors:** Chunsheng Huang, Qizhen Tong, Qiaoling Tong

**Affiliations:** 1 Department of Anesthesiology, Ningbo Medical Center Lihuili Hospital, Medical School of Ningbo University, Ningbo, Zhejiang, China; 2 Department of Operating Room, Ningbo Yinzhou District Integrated Hospital of Traditional Chinese and Western Medicine, Ningbo, China; 3 Department of Operating Room, The Affiliated People’s Hospital of Ningbo University, Ningbo, China; 4 Department of Otolaryngology, Ningbo NO.2 Hospital, Ningbo, Zhejiang, China; Sai Gosavi Specialty Clinic / Nano Hospitals Bangalore / Saraswati Specialty Clinic, INDIA

## Abstract

**Background:**

Chronic pain has emerged as a significant global public health concern. Hence, it is imperative to acquire a more comprehensive comprehension of these characteristics in the adult population of the United States in order to facilitate the development of effective interventions. The objective of this study is to ascertain the prevalence of chronic pain among people in the United States and investigate its association with C-reactive protein (CRP) levels.

**Methods:**

The present study employed a cross-sectional design and utilized data from three cycles of the National Health and Nutrition Examination Survey (NHANES). The study aimed to investigate the relationship between chronic pain status, CRP levels, and potential confounding factors. The study incorporated individuals who successfully fulfilled chronic questionnaires and had CRP assays. Weighted univariate and multivariate logistic regression analyses were performed to examine the correlation between chronic pain and CRP levels. To explore the non-linear relationship, weighted restricted cubic spline (RCS) with three knots coupled with a weighted logistic regression model to assess the dose-response relationship between CRP (continuous variables) and chronic pain.

**Results:**

A total of 10,680 (Weighted 250,814,660.8) adult participants with complete information were included in the analysis and 2612 (Weighted 67978784.88, 27.1%) subjects met the definition of chronic pain. Compared with participants without chronic pain, those with chronic pain had a higher CRP level (*P* < 0.001). The results of the multivariable adjusted logistic regression model suggested that the highest CRP quartile (CRP >  0.52 mg/dL) was associated with a 32% increase in the risk of chronic pain compared with the lowest CRP quartile (CRP ≤  0.09 mg/dL). The RCS result showed that the OR of chronic pain and CRP displayed a linear relationship (*P* = 0.027, Non-linear P = 0.541).

**Conclusions:**

The study found a significant correlation between CRP levels and the presence of chronic pain among people in the United States. Individuals exhibiting elevated levels of CRP demonstrated a heightened propensity for experiencing chronic pain in comparison to individuals with lower CRP levels. Additional investigation is necessary to explore the presence of a causal association between the two variables, as well as the potential underlying mechanisms.

## Introduction

Chronic pain presents a notable public health dilemma, exerting a profound influence on the well-being of countless individuals globally and imposing a considerable strain on healthcare systems [1,2]. Numerous studies have indicated that the development of chronic pain is influenced by a variety of factors, including genetics, socioeconomic level, lifestyle choices (such as smoking, alcohol use, daily physical activity, and nutritional status), as well as occupational characteristics [3–5]. Nevertheless, the management of chronic pain continues to pose a challenging issue. Hence, the intricate complexity of chronic pain mandates a thorough comprehension of its fundamental mechanisms in order to formulate efficacious measures for prevention and treatment.

In recent times, there has been an increasing inclination towards investigating the correlation between inflammatory mechanisms and persistent pain. Notably, C-reactive protein (CRP) has emerged as a significant biomarker indicative of systemic inflammation [6,7]. CRP, an acute-phase reactant synthesized by the liver in reaction to inflammatory stimuli, has been the subject of substantial investigation in relation to several chronic ailments, including cardiovascular disease, diabetes, and autoimmune illnesses [8–11]. Although the involvement of inflammation in the development of these disorders has been suggested, its impact on chronic pain is still being actively studied. An increasing body of scholarly evidence indicates that inflammatory mechanisms may have a role in the onset, worsening, or persistence of chronic pain conditions [12–14]. A prior investigation has established that CRP holds promise as a viable biomarker for chronic pain assessment [15,16]. Nevertheless, there is still uncertainty over whether chronic pain issues are directly linked to systemic inflammation or if they are indicative of the simultaneous existence of sociodemographic, psychological, and lifestyle factors, as well as medical comorbidities.

Notwithstanding the growing body of evidence that establishes a connection between inflammation and chronic pain, there is a scarcity of extensive, population-based investigations that thoroughly examine this association, particularly in regard to the heterogeneous and diverse population of the United States. To address this deficiency, we utilized data from the National Health and Nutrition Examination Survey (NHANES) to conduct a nationwide cross-sectional study that sought to clarify the relationship between CRP levels and the prevalence of chronic pain among adults in the United States.

The motivation to examine this correlation is rooted in the possible ramifications it may have on clinical practice and public health. Gaining insight into the dynamic relationship between chronic pain and inflammatory markers, such as CRP, could facilitate the creation of targeted interventions that regulate inflammatory processes, thereby presenting innovative approaches to the management of pain. Furthermore, by identifying subpopulations that are more susceptible to this association, customized preventive measures can be developed.

## Methods

### Study population

A nationally representative sample of the U.S. populace, the NHANES provides comprehensive data on the health and nutrition of the general population [17]. The NHANES survey data are accessible to the general public, including data researchers and consumers. Data collection at the National Center for Health Statistics (NCHS) occurs biennially. By undertaking a series of interviews (encompassing health-related inquiries, physical examinations, and laboratory tests in addition to demographic, socioeconomic, and dietary inquiries) every two years, the survey generates data that is representative of the entire nation. Due to the restricted availability of the primary outcome (chronic pain) in our study during three consecutive cycles (1999–2000, 2001–2002, and 2003–2004), we restricted our analysis to adult datasets spanning from 1999 to 2004. These individuals had to be at least 20 years old and had completed the Miscellaneous Pain Questionnaire, during which the CRP level in whole blood was measured. All participants furnished written informed consent, and the NCHS Research Ethics Review Board granted approval for the survey protocol. Additional details regarding the NHANES can be found at http://www.cdc.gov/nhanes.

### Other covariates

On the basis of literature review and our clinical experience, we considered the following variables as potential confounders of the relationship between the CRP level and chronic pain: Age, gender (male and female) and race (Mexican American, non-Hispanic Black, non-Hispanic White, other Hispanic, and other/multiracial). According to the WHO criteria, a BMI below 18.5 kg/m² is classified as underweight, while a BMI between 18.5 and 24.9 kg/m² falls within the normal weight range. Individuals with a BMI between 25 and 29.9 kg/m² are considered overweight, and those with a BMI of 30 kg/m² or greater are classified as obese. Other confounding factors include education level (less than high school, and more than high school), marital status (married or living with others, others), insurance, poverty income ratio (PIR), alcohol intake (non-drinker, 1–5 drinks/month, 5–10 drinks/month, and 10 + drinks/month), smoking status (never, current smoker, and former smoker), daily physical activity, health conditions, and laboratory index. The self-reported daily physical activity contained four categories: mainly sit (sitting most of the day), walk around (walking around but no lifting or carrying), light load (lifting light loads and climbing stairs or hills), or heavy load (heavy work and carrying heavy loads). The Prescription Medications Questionnaire was used to gather information on the use of medications, including opioids, non-steroidal antiinflammatory drugs (NSAIDs), and statins, prior to the interview date. S1-3 Tables provide a comprehensive list of the generic names and NHANES codes for each drug category. Health conditions included diabetes, stroke, lung disease, congestive heart failure (CHF), cardiovascular disease (CVD), and cancer or malignancy. Laboratory index included white blood cells (WBC), red blood cells (RBC), hemoglobin (HGB), platelets (PLT), glycosylated hemoglobin (Gly), and brain natriuretic peptide (BNP).

### Determination of CRP

For analysis, blood samples were transported from the Division of Environmental Health Laboratory Sciences, National Center for Environmental Health, CDC, after undergoing processing, storage, and transportation. The quality control and assurance protocols of the NHANES complied with the requirements of the Clinical Laboratory Improvement Act of 1988. Comprehensive guidelines for the collection and processing of specimens were outlined in the NHANES Laboratory/Medical Technologists Procedures Manual.

### Outcome variable

Chronic pain experienced by the participant constituted the outcome variable. This research investigated chronic pain by utilizing the MPQ100 (indicating the extent to which participants experienced pain lasting over 24 hours in the previous month) and MPQ110 (comprising the duration of pain) scores. Chronic pain was delineated in the 11th edition of the International Classification of Diseases (ICD-11) as pain that was either persistent or recurrent in nature, with a duration exceeding three months [18].

### Statistical analysis

The complex sampling methodology involves stratified, cluster, and multistage sampling, in addition to unequal probability sampling that is proportional to a measure of size (PPS). This method requires the incorporation of sampling weights. The design also facilitates the inclusion of additional cycles, which enhances statistical reliability. However, traditional regression techniques are insufficient, as they may produce misleading inferential results. In particular, the standard error and confidence intervals for parameter estimates can be significantly underestimated, leading to an increased likelihood of Type I errors in hypothesis testing. Therefore, we utilized SURVEYMEANS, SURVEYREG, and SURVEYLOGISTIC to conduct precise statistical descriptions and logistic regression analyses that take the complex sampling designs into account. Continuous variables are reported as weighted medians with interquartile ranges (IQRs), while categorical variables are presented as weighted counts and percentages. Group comparisons were conducted using the χ² test for categorical variables and the Mann-Whitney U test for continuous variables, as appropriate. In this study, missing covariates were handled through multiple imputation techniques to minimize bias and maintain statistical power. Specifically, we used the multivariate imputation by chained equations (MICE) method, which assumes that missing data are missing at random (MAR). This approach allows for the imputation of missing values based on observed relationships between covariates. The CRP was classified into four distinct categories. Analysis of the relationship between CRP and chronic pain was performed using multivariate logistic regressions. In the rudimentary Model 1, no adjustments were made for confounding variables. Age and gender were modified in Model 2. Apart from the variables that were modified using Model 2, additional potential confounding factors were also accounted for. All statistical analyses were conducted utilizing R software, specifically version 4.1.1. A significance level of *P* <  0.05 was applied to each analysis.

## Results

### Description of the study population

**Fig 1:**
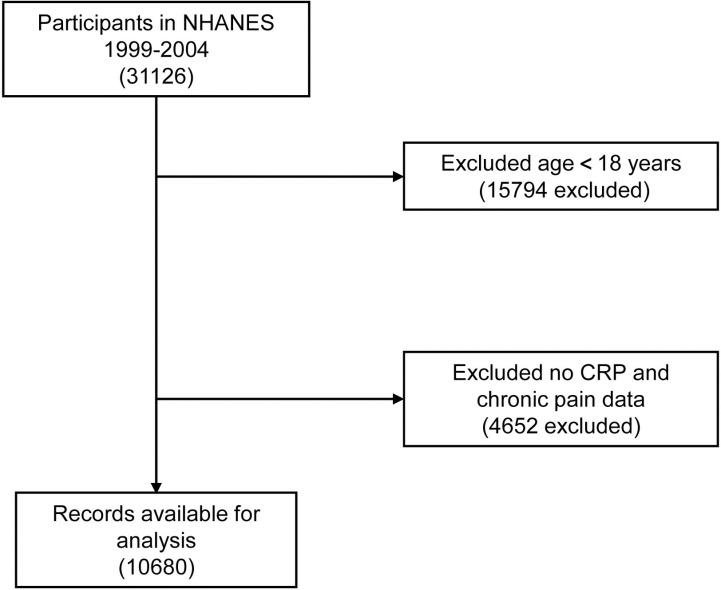
Screening of admissions for inclusion.

This study included 10,680 (Weighted 250,814,660.8) participants aged at least one year who had CRP level and chronic pain status measurements (Fig 1). Of the included participants, the median age of the study patients was 44.00 (32.00, 56.00) years old, and the gender distribution was relatively equal. The median CRP of the study patients was 0.23 (0.09-0.52) mg/dL. Participants with chronic pain made up more than a fifth of the study population (Table 1). Gender, race, BMI, education level, PIR, smoking status, diabetes, stroke, lung disease, CHF, CVD, cancer or malignancy, opioids use, NSAIDs use, statins use, WBC, RBC, PLT, BNP and CRP were significantly different in the chronic pain group compared with the non-chronic pain group (*P* < 0.05, Table 1). Participants with chronic pain were more likely to be female, non-Hispanic White, and obese. They also tended to have an education level beyond high school, a lower PIR, and a history of being current smokers. Additionally, these participants had a history of diabetes, stroke, lung disease, CHF, CVD, and cancer or malignancy. They had a higher percentage of individuals taking opioids, NSAIDs, and statins, along with elevated levels of WBC, PLT, BNP, and CRP. Conversely, they exhibited lower levels of RBC and HGB.

**Table 1:** Baseline characteristics of subjects.

Covariates	Total (n = 10680)	No chronic group (n = 8068)	Chronic group (n = 2612)	*P*
**Weighted number**	250814660.8	182835876	67978784.88	
**Age**	44.00 (32.00, 56.00)	43.00 (32.00, 57.00)	44.00 (34.00, 55.00)	0.233
**Gender**				0.004
Female	129925453.7 (51.8%)	92741139.3 (50.7%)	37184314.5 (54.7%)	
Male	120889207.1 (48.2%)	90094736.7 (49.3%)	30794470.4 (45.3%)	
**Race**				< 0.001
Mexican American	17673354.4 (7.0%)	14617587.8 (8.0%)	3055766.6 (4.5%)	
Non-Hispanic Black	23673322.8 (9.4%)	17966487.5 (9.8%)	5706835.3 (8.4%)	
Non-Hispanic White	183162250.5 (73.0%)	130473133.7 (71.4%)	52689116.8 (77.5%)	
Other Hispanic	15061307.3 (6.0%)	11155690.1 (6.1%)	3905617.3 (5.7%)	
Other/multiracial	11244425.7 (4.5%)	8622976.9 (4.7%)	2621448.8 (3.9%)	
**BMI**				< 0.001
Underweight	4625855.7 (1.8%)	3229575.2 (1.8%)	1396280.5 (2.1%)	
Normal weight	81636718.3 (32.5%)	62588411.5 (34.2%)	19048306.8 (28.0%)	
Overweight	87162430.3 (34.8%)	64471372.5 (35.3%)	22691057.8 (33.4%)	
Obesity	77389656.6 (30.9%)	52546516.8 (28.7%)	24843139.8 (36.5%)	
**Education level**				0.039
Less than high school	114038738.3 (45.5%)	81614554.7 (44.6%)	32424183.6 (47.7%)	
More than high school	136775922.5 (54.5%)	101221321.3 (55.4%)	35554601.2 (52.3%)	
**Marital Status**				0.393
Married or living with others	164731017.5 (65.7%)	119519793.8 (65.4%)	45211223.7 (66.5%)	
Others	86083643.4 (34.3%)	63316082.2 (34.6%)	22767561.2 (33.5%)	
**Insurance**	207026385.4 (82.5%)	150674769.2 (82.4%)	56351616.2 (82.9%)	0.708
**PIR**	3.01 (1.51, 4.98)	3.06 (1.60, 5.00)	2.84 (1.34, 4.64)	< 0.001
**Alcohol intake**				0.421
Non-drinker	115829113.6 (46.2%)	83932737.6 (45.9%)	31896376.0 (46.9%)	
1–5 drinks/month	78518331.4 (31.3%)	58185217.3 (31.8%)	20333114.0 (29.9%)	
5–10 drinks/month	19114857.3 (7.6%)	14089577.7 (7.7%)	5025279.6 (7.4%)	
10+ drinks/month	37352358.5 (14.9%)	26628343.3 (14.6%)	10724015.3 (15.8%)	
**Smoking status**				< 0.001
Never	196260326.3 (78.2%)	146231555.7 (80.0%)	50028770.7 (73.6%)	
Current smoker	52076515.5 (20.8%)	34644761.9 (18.9%)	17431753.7 (25.6%)	
Former smoker	2477819.0 (1.0%)	1959558.4 (1.1%)	518260.6 (0.8%)	
**Daily physical activity**				0.118
Main sit	61910800.8 (24.7%)	43975264.8 (24.1%)	17935536.0 (26.4%)	
Walk around	126603341.8 (50.5%)	94092339.7 (51.5%)	32511002.1 (47.8%)	
Light load	43715972.1 (17.4%)	31647698.8 (17.3%)	12068273.3 (17.8%)	
Heavy load	18584546.1 (7.4%)	13120572.6 (7.2%)	5463973.5 (8.0%)	
**Diabetes**	15912178.5 (6.3%)	10146929.3 (5.5%)	5765249.2 (8.5%)	< 0.001
**Stroke**	5433095.5 (2.2%)	3271828.6 (1.8%)	2161266.9 (3.2%)	< 0.001
**Lung disease**	19671566.8 (7.8%)	11180118.6 (6.1%)	8491448.2 (12.5%)	< 0.001
**CHF**	5419178.7 (2.2%)	3494546.8 (1.9%)	1924631.9 (2.8%)	0.020
**CVD**	8567229.0 (3.4%)	5546093.3 (3.0%)	3021135.6 (4.4%)	0.002
**Cancer or malignancy**	19906383.1 (7.9%)	13420531.9 (7.3%)	6485851.3 (9.5%)	0.013
**Drug use**				
**Opioids**	12306263.0 (4.9%)	3752884.7 (2.1%)	8553378.3 (12.6%)	< 0.001
**NSAIDs**	18566569.6 (7.4%)	9043697.3 (4.9%)	9522872.3 (14.0%)	< 0.001
**Statins**	22557981.0 (9.0%)	15693453.5 (8.6%)	6864527.5 (10.1%)	0.037
**WBC ( × 10**^**9**^**/L)**	6.90 (5.70, 8.40)	6.90 (5.70, 8.30)	7.10 (5.80, 8.60)	0.013
**RBC ( × 10**^**9**^**/L)**	4.72 (4.39, 5.07)	4.73 (4.39, 5.09)	4.70 (4.39, 5.03)	0.046
**HGB (g/dL)**	14.50 (13.60, 15.50)	14.50 (13.60, 15.50)	14.50 (13.50, 15.40)	0.060
**PLT ( × 10**^**9**^**/L)**	261.00 (224.00, 306.00)	260.00 (223.00, 304.00)	263.00 (225.43, 313.00)	0.016
**Gly (%)**	5.30 (5.10, 5.50)	5.30 (5.10, 5.50)	5.30 (5.10, 5.60)	0.235
**BNP (mg/mL)**	45.66 (22.13, 93.03)	44.67 (21.58, 92.22)	48.46 (23.45, 95.57)	0.007
**CRP (mg/dL)**	0.20 (0.08, 0.46)	0.19 (0.08, 0.43)	0.23 (0.10, 0.54)	< 0.001

BMI: body mass index, PIR: poverty income ratio, CHF: congestive heart failure, CVD: cardiovascular disease, NSAIDs: Non-steroidal antiinflammatory drugs, WBC: white blood cells, RBC: red blood cells, HGB: hemoglobin, PLT: platelets, Gly: glycosylated hemoglobin, BNP: brain natriuretic peptide, CRP: C-reactive protein.

### Association of CPR with chronic pain

Of the participants included in the study, 2443 (weighted 67025132.7) participants had a CRP <  0.09 mg/dL (Q1 group), 2782 (weighted 66749171.9) participants were between 0.09 and 0.23 mg/dL (Q2 group), 2733 (weighted 61163243.4) participants were between 0.23 and 0.52 mg/dL (Q3 group), and 2722 (weighted 55877112.8) patients were ≥  0.52 mg/dL (Q4 group). The incidence of chronic pain was significantly higher among patients with Q4 group (26.2%), compared with patients with Q3 group (24.9%), Q2 group (25.9%), and Q1 group (22.9%; *P* < 0.001, Table 2). We have used three multivariate logistic regression models to show the relationship between CRP level with chronic pain in Table 3: model 1, no covariate was adjusted; model 2, age, and gender were adjusted; model 3, age, gender, race, BMI, education level, PIR, smoking status, diabetes, stroke, lung disease, CHF, CVD, cancer or malignancy, opioids use, NSAIDs use, statins use, WBC, RBC, PLT, and BNP were adjusted. We found a significantly positive association between CRP level with the incidence of chronic pain in the unadjusted model (Model 1). In model 2, the ORs (95% CIs) after adjusting for age and gender for incidence of chronic pain in participants with Q2 group, Q3 group and Q4 group compared with those with Q1 group were 1.19 (1.00–1.42), 1.27 (1.03–1.57) and 1.53 (1.27–1.84), respectively (Table 3, *P* <  0.05). In model 3, the ORs (95% CIs) after adjusting for related indexes for incidence of chronic pain in participants with Q4 group compared with those with Q1 group was 1.32 (1.10–1.58) (Table 3, *P* <  0.001). For sensitivity analysis, different CRP levels group were transformed into a categorical variable, and the *P* value for the trend of different CRP levels group with categorical variables was consistent with the result of different CRP levels group as a continuous variable in the different models (Table 3, *P*_trend_ <  0.05).

**Table 2:** Individuals with/without chronic pain by CRP quartiles.

CRP quartiles (mg/dL)		Individuals			*P*
**Quartiles**	**Range**	**Total sample**	**No chronic pain**	**Chronic pain**	< 0.001
1	0.01–0.09	67025132.7	51452650.3 (28.1%)	15572482.4 (22.9%)	
2	0.09–0.23	66749171.9	49124920.1 (26.9%)	17624251.9 (25.9%)	
3	0.23–0.52	61163243.4	44207091.3 (24.2%)	16956152.1 (24.9%)	
4	0.52–29.60	55877112.8	38051214.3 (20.8%)	17825898.5 (26.2%)	

CRP: C-reactive protein

**Table 3:** Logistic regression model of the effect of different levels of CRP on chronic pain.

Groups	β	SE	Wald	OR (95%CI)	*P*	*P* for trend
Model 1^a^						< 0.001
Q1				1.00		
Q2	0.170	0.085	3.963	1.19(1.00–1.41)	0.053	
Q3	0.237	0.101	5.474	1.24(1.09–1.41)	0.024	
Q4	0.437	0.089	23.892	1.40(1.23–1.59)	< 0.001	
Model 2^b^						< 0.001
Q1				1.00		
Q2	0.178	0.087	4.152	1.19(1.00–1.42)	0.048	
Q3	0.240	0.103	5.356	1.27(1.03–1.57)	0.026	
Q4	0.423	0.092	21.079	1.53(1.27–1.84)	< 0.001	
Model 3^c^						0.017
Q1				1.00		
Q2	0.158	0.082	3.648	1.17(0.98–1.39)	0.072	
Q3	0.181	0.098	3.429	1.20(0.98–1.47)	0.081	
Q4	0.275	0.086	10.240	1.32(1.10–1.58)	0.005	

^a^Unadjusted.

^b^Adjusted for age, and gender.

^c^Adjusted for age, gender, race, BMI, education level, PIR, smoking status, diabetes, stroke, lung disease, CHF, CVD, cancer or malignancy, opioids use, NSAIDs use, statins use, WBC, RBC, PLT, and BNP.

### The dose-response relationship of CRP and the risk of chronic pain

**Fig 2:**
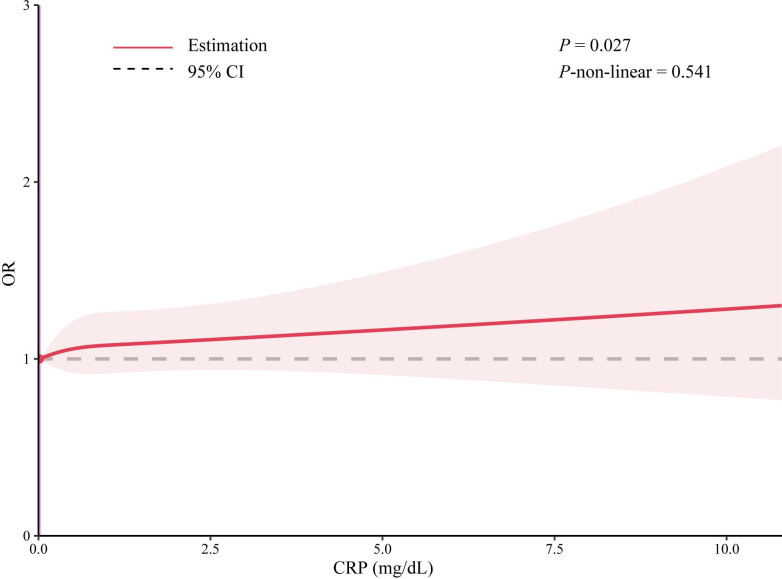
Dose–response relationship between CRP and chronic pain.

A dose-response relationship was examined between CRP and the risk of chronic pain. The result showed that the OR of chronic pain and CRP displayed a linear relationship (*P* = 0.027, Non-linear *P* = 0.541), as shown in Fig 2.

## Discussion

Using data from the NHANES, the current nationwide cross-sectional study examined the association between CRP levels and chronic pain in a representative sample of US adults. Chronic pain was 32% more likely to affect individuals in the highest CRP quartile than those in the lowest; a dose-response relationship existed between CRP level and the probability of chronic pain. The results of our study demonstrate a statistically significant correlation between increased CRP levels and the incidence of chronic pain, providing insight into the possible involvement of inflammation in relation to this widespread health issue.

In 2016, the CDC estimated that 20.4% of adults in the United States suffered from chronic pain [19]. During 1999–2004, 24.5% of the US population reported having self-reported chronic pain, according to this study. This finding suggests that the chronic pain questionnaire was administered exclusively to participants who were at least 20 years old, resulting in an age distribution that did not include adults younger than 20 years old. Consequently, the proportion of individuals with chronic pain was marginally higher [20,21]. Chronic pain undermines the quality of life and disrupts individuals’ daily routines. Opioids may be necessary for patients with chronic pain, thereby increasing the risk of opioid misuse, abuse, and addiction, as well as adverse events such as constipation, cognitive impairment, respiratory suppression, and mortality [22,23].

There are several credible hypotheses regarding the fundamental mechanisms that link CRP to chronic pain, though these mechanisms remain largely enigmatic. Nociceptive (resulting from damage to tissues), neuropathic (caused by damage to nerves), or nociplastic (arising from a sensitized nervous system) stimuli may induce chronic pain [24,25]. Instances of chronic pain frequently manifest inflammatory mechanisms, even in the absence of a traditional inflammatory diagnosis. The activation of the immune system occurs in reaction to tissue damage or injury, resulting in the secretion of a multitude of inflammatory mediators [26–28]. An increase in CRP levels could potentially lead to the sensitization of nociceptive pathways, which play a crucial role in the transmission of pain signals [29]. The process of sensitization may lead to an intensified reaction to harmful stimuli, thereby playing a role in an elevated perception of pain [30]. The reciprocal exchange of information between the nervous system and immune system is of paramount importance in regulating pain. Cytokines and other signaling molecules secreted by immune cells have the capacity to exert direct influence on neurons, thereby modulating their sensitivity to pain stimuli [31–33]. These neuroimmune interactions may involve CRP, thereby contributing to the maintenance of chronic pain states [34]. It is critical to acknowledge that although an association has been observed between CRP and chronic pain, establishing a causal relationship remains difficult. Chronic pain is a complex phenomenon that is impacted by a variety of genes, the environment, and psychosocial elements. Chronic pain may not be the etiology of elevated CRP levels; conversely, both chronic pain and elevated CRP levels could indicate the presence of an underlying inflammatory process.

The implications of the study’s results for the prevention and treatment of chronic pain in adults are substantial. Screening for CRP levels could be a straightforward and cost-effective method for identifying individuals at increased risk for chronic conditions. Anti-inflammatory agents, lifestyle adjustments, and individualized treatment strategies customized for patients with elevated CRP levels could potentially serve as innovative approaches to enhance pain management. Nonetheless, it is necessary to acknowledge a number of limitations of this investigation. Initially, the cross-sectional design of the study precludes us from establishing a causal relationship between CRP level and chronic pain. In order to investigate the temporal relationship between these variables in greater depth, longitudinal studies are necessary. Secondly, the data used in this study dates back 20 years, covering the period from 1999 to 2004. Over the past two decades, various factors impacting the prevalence of chronic pain have shifted significantly, particularly with the notable rise in inflammation-related conditions. While the findings from this time frame remain valuable and provide key insights, future research should focus on more recent data to better reflect current trends and contributing factors. Therefore, further experimental research is needed to explore more precise methods of assessment. Furthermore, the assessment of chronic pain was conducted using self-reported questionnaires, which are inherently prone to recall bias and subjectivity. Subsequent investigations that incorporate objective metrics for chronic pain would produce more robust findings. Similar to any observational study, it is impossible to completely rule out the existence of unmeasured confounding variables.

## Conclusion

In summary, our inquiry establishes a noteworthy association between the concentration of CRP and the presence of chronic pain. A dose-response correlation was observed between CRP level and the probability of developing chronic pain. Although causation has yet to be established, these results add to the expanding corpus of research that establishes a link between inflammation and chronic pain. Additional research, encompassing mechanistic inquiries and prospective studies, is imperative in order to elucidate the intricacies of this correlation, provide guidance for precise interventions, and propel our comprehension of the intricate relationship between inflammation and chronic pain forward.

## Supporting information

S1 Table Drug code for opioids. (PDF)

S2 Table Drug code for NSAIDs. (PDF)

S3 Table Drug code for statins. (PDF)
