# Supplementary material for: Association between C-reactive protein and chronic pain in US adults: A nationwide cross-sectional study
Source: PLoS One. 2025 Feb 7;20(2):e0315602. doi: 10.1371/journal.pone.0315602 (PMC11805396; doi:10.1371/journal.pone.0315602)
Supplement: S1 Table — (PDF) [file pone.0315602.s001.pdf]

S1 Table. Drug code for opioids

| Drug code | Drug name |
|---|---|
| d00012 | CODEINE; |
| d00017 | MEPERIDINE; |
| d00334 | PENTAZOCINE; |
| d00360 | PROPOXYPHENE; |
| d03826 | TRAMADOL |
| d03423 | ACETAMINOPHEN; CODEINE |
| d03424 | ASPIRIN; CODEINE |
| d03425 | ACETAMINOPHEN; BUTALBITAL; CAFFEINE; CODEINE |
| d03426 | ASPIRIN; BUTALBITAL; CAFFEINE; CODEINE |
| d03430 | ASPIRIN; CAFFEINE; DIHYDROCODEINE |
| d04269 | ACETAMINOPHEN; CAFFEINE; DIHYDROCODEINE |
| d03433 | MEPERIDINE; PROMETHAZINE |
| d03434 | MEPERIDINE; PROMETHAZINE |
| d03435 | MEPERIDINE; PROMETHAZINE |
| d03676 | NALOXONE; PENTAZOCINE |
| d03682 | ACETAMINOPHEN; PENTAZOCINE |
| d04766 | ACETAMINOPHEN; TRAMADOL |
| d00308 | MORPHINE; |
| d03075 | HYDROCODONE; |
| d07453 | TAPENTADOL; |
| d00824 | OPIUM |
| d03428 | ACETAMINOPHEN; HYDROCODONE |
| d03429 | ASPIRIN; HYDROCODONE |
| d03436 | BELLADONNA; OPIUM |
| d04225 | HYDROCODONE; IBUPROFEN |
| d00233 | FENTANYL; |
| d00329 | OXYCODONE; |
| d00833 | OXYMORPHONE; |
| d00255 | HYDROMORPHONE |
| d03431 | ACETAMINOPHEN; OXYCODONE |
| d03432 | ASPIRIN; OXYCODONE |
| d00050 | METHADONE |
| d00840 | BUPRENORPHINE |
| d04819 | BUPRENORPHINE; NALOXONE |
