# Supplementary material for: Association between C-reactive protein and chronic pain in US adults: A nationwide cross-sectional study
Source: PLoS One. 2025 Feb 7;20(2):e0315602. doi: 10.1371/journal.pone.0315602 (PMC11805396; doi:10.1371/journal.pone.0315602)
Supplement: S2 Table — (PDF) [file pone.0315602.s002.pdf]

S2 Table. Drug code for NSAIDs

Drug code	Drug name
d00170	ASPIRIN
d03458	ASPIRIN; BUTALBITAL
d03457	ASPIRIN; BUTALBITAL; CAFFEINE
d03426	ASPIRIN; BUTALBITAL; CAFFEINE; CODEINE
d03430	ASPIRIN; CAFFEINE; DIHYDROCODEINE
d03472	ASPIRIN; CAFFEINE; ORPHENADRINE
d03435	ASPIRIN; CAFFEINE; PROPOXYPHENE
d03469	ASPIRIN; CARISOPRODOL
d03470	ASPIRIN; CARISOPRODOL; CODEINE
d03424	ASPIRIN; CODEINE
d04497	ASPIRIN; DIPYRIDAMOLE
d03429	ASPIRIN; HYDROCODONE
d03448	ASPIRIN; MEPROBAMATE
d03468	ASPIRIN; METHOCARBAMOL
d03432	ASPIRIN; OXYCODONE
d03291	ASPIRIN; PSEUDOEPHEDRINE
d00170	ASPIRIN
d03458	ASPIRIN; BUTALBITAL
d03457	ASPIRIN; BUTALBITAL; CAFFEINE
d00015	IBUPROFEN
d00019	NAPROXEN
d07130	NAPROXEN; SUMATRIPTAN
d00039	INDOMETHACIN
d04380	CELECOXIB
d00848	DICLOFENAC
d03213	DICLOFENAC OPHTHALMIC
d04722	DICLOFENAC TOPICAL
d04271	DICLOFENAC; MISOPROSTOL
d04532	MELOXICAM
d00028	KETOPROFEN
d00851	ETODOLAC
d00343	PIROXICAM
d00033	SULINDAC
d00853	OXAPROZIN
d00026	FENOPROFEN
d00239	FLURBIPROFEN
d00273	KETOROLAC
d03214	KETOROLAC OPHTHALMIC
d00054	TOLMETIN
d00842	SALSALATE
d00283	MECLOFENAMATE
d00208	DIFLUNISAL

d00310	NABUMETONE
c00061	NONSTEROIDAL ANTI-INFLAMMATORY AGENTS - UNSPECIFIED
