# Supplementary material for: Association between C-reactive protein and chronic pain in US adults: A nationwide cross-sectional study
Source: PLoS One. 2025 Feb 7;20(2):e0315602. doi: 10.1371/journal.pone.0315602 (PMC11805396; doi:10.1371/journal.pone.0315602)
Supplement: S3 Table — (PDF) [file pone.0315602.s003.pdf]

S3 Table. Drug code for statins

| Drug code | Drug name |
|---|---|
| d00746 | SIMVASTATIN |
| d07805 | SIMVASTATIN; SITAGLIPTIN |
| d05048 | AMLODIPINE; ATORVASTATIN |
| d04105 | ATORVASTATIN |
| d04140 | CERIVASTATIN |
| d05348 | EZETIMIBE; SIMVASTATIN |
| d03183 | FLUVASTATIN |
| d00280 | LOVASTATIN |
| d04787 | LOVASTATIN; NIACIN |
| d07110 | NIACIN; SIMVASTATIN |
| d07637 | PITAVASTATIN |
| d00348 | PRAVASTATIN |
| d04851 | ROSUVASTATIN |
